# Telenursing implications for future education and practice: Nursing students’ perspectives and knowledge from a course on child health

**DOI:** 10.1371/journal.pone.0294711

**Published:** 2023-11-27

**Authors:** Omar Mohammad Ali Khraisat, Ahmad M. Al-Bashaireh, Eman Alnazly

**Affiliations:** 1 Faculty of Nursing, Al-Ahliyya Amman University, Amman, Jordan; 2 Faculty of Health Science, Higher Colleges of Technology, Fujairah, United Arab Emirates; 3 Nursing Faculty, Al-Ahliyya Amman University, Amman, Jordan; Qatar University College of Nursing, QATAR

## Abstract

**Background:**

Technology advancements have altered the standard of nursing care, and education. This suggests the necessity to equip prospective nurses to provide competent care in a highly technical and digital environment via telenursing.

**Aims:**

The aim of this study was to assess the perceptions and knowledge of nursing students about telenursing.

**Methods:**

Using a descriptive cross-sectional design. A self-reported questionnaire was used. The distribution of 110 questionnaires to nursing students attending two private colleges in Jordan resulted in an 83.6% (n = 92) response rate. Descriptive and inferential statistics were applied.

**Results:**

The results showed a positive perception toward telenursing practice, as well as the difficulty in precisely defining telenursing. Age, internet usage patterns, and knowledge were all factors considered telenursing predictors. 32% of the variance was explained by the model. Telenursing would be beneficial for future nursing professionals by incorporating telenursing into the curriculum.

**Conclusions:**

The learning environment is extends somewhat beyond the classroom, making it is necessary to integrate telenursing into education in order to redefine the future of the nursing practice.

## Introduction

The rapid development of information and communication technology has altered the nursing care paradigm and made telenursing a reality [1, 2]. With the introduction of telenursing, new changes are anticipated in the fundamental nursing domains of "person," "environment," "nursing," and "health" [1]. The provision of nursing care, patient education, and support services using telecommunications technology is known as telenursing [3]. Information and communication technology (ICT) advancements have given nurses new resources and platforms to connect with patients remotely, evaluate their health, impart knowledge, and offer assistance [4].

The delivery of healthcare has been altered via telenursing [3]. Wearable technology and sensors in conjunction with telenursing enable remote monitoring of patients’ vital signs, adherence to medication, and general well-being [4]. This reduces the need for hospital readmissions, improves patient outcomes, and gets beyond geographic limitations by enabling the early detection and timely action of changes in health conditions [5]. Furthermore, patients might be empowered to actively participate in their own healthcare by increasing the accessibility of educational materials through telenursing [5, 6]. By enabling simple communication and collaboration among medical specialists, it promotes the continuity of care [7–9].

Also, in order to creatively prepare for this rapidly growing field, it is crucial to understand how nursing students view telenursing in this hyperconnected world [1]. Nursing professionals need to acquire new skills and methods in order to practice telenursing [2–5]. Numerous studies have highlighted the positive impacts of telenursing being included in nursing courses [6–12]. If nurses are not proficient in technology, it could affect the quality and safety of the care they provide [13].

Further, there are several concerns regarding ethics with telenursing that need to be addressed. Numerous legal and ethical questions are raised by telenursing [8, 10]. Since patient information is conveyed electronically, nurses are responsible for ensuring compliance with privacy and confidentiality laws. The ethical and regulatory guidelines governing telenursing in their practice environments must be understood by nurses [8, 10].

Some of the ethical issues unique to telenursing were as follows: sensitive patient data is transmitted over electronic platforms during telenursing. It is essential to protect patients’ privacy and the confidential nature of their health information. Legal and moral requirements for data protection, encryption, and safe patient data storage must be followed by nurses. Obtaining informed consent is another crucial ethical concept in the field of healthcare [12]. In telenursing, it’s crucial to make sure that patients comprehend everything there is to know about remote care [11, 12]. Nonetheless, there are ethical concerns regarding the lack of digital communication and universal access to technology. It’s possible that some patients lack access to stable internet service or the necessary equipment to participate in telenursing [10].

Nurses must be aware of these differences and work toward fair healthcare delivery to prevent increasing marginalization of vulnerable communities [14, 15]. Effective nursing care depends on the nurse and the patient developing a therapeutic relationship [16, 17]. By carefully listening to patients and addressing their emotional and psychological needs, nurses must work to establish a positive and compassionate virtual environment [18]. Nurses must be conscious of their limitations and, when necessary, swiftly refer patients to the proper healthcare facilities or professionals [19].

For nursing students, telenursing is very important [18]. It gives nursing students the chance to connect with patients outside of traditional healthcare settings and participate in clinical experiences [1, 20, 21]. By gaining experience and knowledge in this field, nursing students can position themselves for a variety of roles, such as telehealth nurse, remote patient monitoring specialist, tele triage nurse, or telehealth educator [10, 12]. Students who recognize telenursing during their education can help develop adaptability and resilience, qualities crucial in the rapidly changing healthcare sector [8, 14, 22].

Hence, in Jordan, limited evidence exists to assess the nursing students’ perspectives and telenursing knowledge. This study sought to assess nursing students ’perception, and knowledge about telenursing.

### Literature review

Future education and practice will be affected by telenursing, which is the delivery of nursing care remotely via telecommunications technology [18]. Understanding and utilizing the potential of telenursing can be significantly aided by the perspectives and knowledge of nursing students [12, 13]. The common platforms and telecommunications technologies used in telenursing must be mastered by nursing students [16]. This covers electronic health records, remote monitoring tools, video conferencing, and mobile health software. Effective telenursing practice requires an understanding of the potential and constraints of these technologies [19].

In telenursing, effective communication abilities are crucial [18]. When communicating remotely, nursing students should practice building rapport, expressing empathy, and giving precise directions [22]. Nurse educators currently have a primary obligation to get students ready for realistic practice [12]. To deliver patient-centered and culturally competent care remotely, nursing students need gain cultural competency abilities. Understanding cultural norms, customs, and health inequities that may influence healthcare delivery is part of this [22]. Less was covered by the undergraduate nursing program in terms of telenursing’s practical applications [14, 15, 23]. It is unclear whether nursing students have received enough training in telenursing for patient care [15].

Further, due to demographic and epidemiological changes including population aging, a rise in chronic diseases and the spread of infectious and contagious diseases, telehealth, and telenursing has become widely used in health services around the world [17, 18, 22, 23]. The expansion of health coverage to remote areas and the lowering of expenditures are two benefits of this care method [19]. in some countries, notably the United States of America (USA) and the United Kingdom (UK), telehealth has shown to be an efficient solution to address the unmet need for safe, routine clinical treatment during the epidemic [24]. In Jordan, COVID-19 pandemic was posing serious healthcare issues as many other countries.

It is challenging to create and implement a national telehealth program in Jordan due to the numerous challenges that exist there [24]. Nursing students can develop insightful perspectives and knowledge to help them get ready for the changing telenursing environment by thinking about these issues. By taking advantage of telenursing technologies and offering patients high-quality remote care, they can help to shape the future of nursing practice.

The anticipated COVID-19 nursing pandemic resolution, which would permit telenursing inclusion in undergraduate curricula, will be a critical milestone in the telenursing scenario globally [10, 13, 15]. Furthermore, in light of the disadvantages of utilizing technologies for nursing care in the event of a pandemic, this resolution setting criteria for digital health has been established to govern the practice of telenursing [17, 18].

For clinical learning requirements, it is crucial to evaluate the nursing student’s opinions on telenursing [13, 15]. Student objectives for clinical can be met by emphasizing inclusiveness, belongingness, and clinical reasoning in addition to case studies or virtual simulations as a teaching method [12]. Riley et al. (2021) address the use of virtual classrooms to offer pediatric courses; modifications include having students participate in unfolding case studies, which had no impact on student performance or fulfillment in contrast to earlier sessions [13].

Since online education has taken the role of the traditional face-to-face on-campus classes, nursing faculty members have been put under strain and have had to be creative in order to address their worries and issues while teaching clinical courses [27]. Other issues were the focus on student relationships, continuity of educational quality, reaching learning goals, and striving for highly calibrated competencies in line with Benner’s paradigm [25–27].

In light of the growing prevalence of telenursing, nursing education schools must include telenursing principles and skills in their curricula. Together with practical instruction on utilizing telecommunications technology and carrying out remote patient assessments, this also includes providing educational knowledge on telenursing concepts, ethics, and legal matters.

Blended learning methodologies can be utilized to incorporate telenursing into nursing education. These methods blend traditional classroom instruction with online learning elements, giving students access to learning resources as well as opportunities to participate in case studies and virtual simulations. Interprofessional education, in which students from several healthcare disciplines (such as nursing, medicine, pharmacy, and social work) train together to develop collaborative abilities, should be emphasized in future nursing education.

## Methods

### Design and setting

A descriptive and cross-sectional design was used. It is expected to be suitable for explaining and understanding new phenomena. Before starting interventional studies, this is a crucial step [28]. The study was conducted among conveniently chosen nursing students from two nursing college from private sector in Amman the capital of Jordan.

### Inclusion and exclusion criteria

All nursing students who were close to graduating and who had to be enrolled in a child health nursing course during their academic year were included in the study; students who were not enrolled were excluded. The transition from undergraduate nursing students to beginning-level registered nurses occurs throughout the third and fourth years of nursing school; for this reason, they are chosen. In this course child and family health is addressed in depth. The fundamentals of health promotion, maintenance, and restoration for children of various ages and their families will be explained to the students through active, hands-on training. This course’s main objective is to teach students how to apply the nursing process, it focuses on the most recent evidence-based nursing practices and how telenursing can be used to care for both healthy and unwell children and their families.

### Sample

A convenience sample was used. Data were gathered from two nursing colleges between 20 July and 30 September 2022. Using Alpha of 0.05, power of 0.8, medium effect size, and regression analysis, a sample size of 98 students was chosen in accordance with Cohen’s (1992) guidelines [28]. Over-sampling can be particularly helpful when trying to improve comprehension and combat participant attrition. In a study or research project, participant attrition is the loss of participants or non-response, which can introduce bias and influence the generalizability of the findings. By expanding the representation of the groups that experienced attrition, oversampling can help offset participant attrition and lessen the impact of the missing data. The response rate for a survey was 83.6% (92 out of 110).

### Measurements

A self-reported questionnaire created by Glinkowski et al. (2013) using Google Docs (https://docs.google.com) was used to gather the data. There were 28 items on this 5-point Likert scale, four of which had negative wording. Participants’ responses ranged from strongly disagreeing (1) to strongly agreeing (5) [9]. The demographic component of the questionnaire included questions about age, gender, access to computers and the internet, how frequently nursing students utilized the internet each day, knowledge of telenursing terminology, and the criteria for telenursing practice that were regarded to be telenursing knowledge. After being translated into Arabic, the questionnaire was translated back into English to evaluate the consistency of the items.

### Pilot study

In order to evaluate the psychometric properties of the instrument, how long it takes to complete, and how easily it is understood a pilot research was carried out at the college of Nursing at the Al-Ahliyya Amman University (AAU). Within five to fifteen minutes, twenty nursing students finished the questionnaire. The reliability of the questionnaire (28 items) showed an alpha coefficient of 0.89. Two academic staff members from AAU with PhDs in nursing informatics examined the questionnaire’s content validity. The study sample did not include the pilot study participants.

### Ethical considerations

The ethical approval was granted by the Scientific Research and Ethical Committee of the Al-Ahliyya Amman University’s Faculty of Nursing (Reference Number (MM 3/7-2022)). The participants received a detailed description of the study’s benefits and risks through written consent. There was no risk, it was told to them. Additionally, the fact that participation was voluntary was made clear to them and that they might discontinue the study at any time without consequences or coercion (for example, their grades wouldn’t be impacted). In addition, participants’ responses were given numbers, the data was stored on a secure computer protected by a password, and it was fully deleted after the study was over, giving them the assurance that all the information acquired would be anonymous. Additionally, the authors gave their consent for the questionnaire to be used.

### Data collection and procedures

The colleges where the study was conducted gave their officials permission. In a classroom context, the participants were approached. All study participants who provided written consent got thorough information about the risks and benefits of the study. The Web-based surveying link was sent to students at the conclusion of class with their instructor’s approval. Participants were provided with the questionnaire link along with a cover letter explaining the purposes of the study. The following statement was added because internet research concerns privacy risks:

"When using the internet to gather information, there is always the chance of interference from an outside source. There is always a chance of hacking or other security breaches that could jeopardize the confidentiality of your responses, even if it will be safeguarded after the data are retrieved from the internet. Please be aware that you are not required to respond to any questions".

The survey’s design requires potential participants to click a "button" or type in a response indicating that they have read the consent/assent information (as outlined in 1 above) and agree to participate. The potential participant will be taken to the research survey questionnaire after selecting the "button." In other words, a participant doesn’t see the survey questions until after he or she clicks on or fills in a response to signify their voluntary involvement.

The Internet Protocol (IP) addresses were removed from the downloaded data file after being acquired by the survey instrument. The online survey’s replies were all removed. There were no identifiers, including IP addresses or other electronic identifiers, in the final data file that was used for data analysis. Additionally, a password-protected personal computer was where the data file was kept. Additionally, the backup data files were kept in a safe place.

### Data analysis

The data were analyzed using SPSS for Windows version 22 (Statistical Package for Social Science). Data were checked for entry errors, missing data, and outliers as well as reverse coding the negatively phrased questions. Using descriptive statistics including frequency, percentage, mean, and standard deviation, the sample’s characteristics were described. Inferential statistics were applied to determining the factors that affected the perception of telenursing. Outliers and missing values were absent. The cutoff for statistical significance was P < 0.05.

## Results

### Characteristics of the sample

A 110 nursing students made up the sample for this study, with an overall response rate of 92 (83.6%), and 58 (63%) of them were male. The mean age of the participants, who range in age from 20 to 30, is 21.8 years. Even while 88% of the participants had access to computers, only about three-quarters of them had smartphones.

Similar to that, nearly all of the participants (97.8%) had access to the internet. Only two of them didn’t have an internet connection. Only 3.3% of individuals reported not using the internet at all, while the majority of participants (53.3%) reported to using it more than three hours each day (Table 1).

**Table 1:** Participants’ demographic characteristics (N = 92).

Variable	Group	Frequency	Percentage
Age M 21.8 years			
SD = 1.75			
R = 20–30			
Gender	Male	58	63
	Female	34	37
Access to computer	No	11	12
	Yes	81	88
	Yes, I use it only in a library	29	31.5
	Smartphone	63	68.5
Access to the internet	Yes	90	97.8
	Yes, only in the library	27	30
	Wi-Fi	63	70
	No	2	2.2
Frequency of using internet	I don’t use it at all	3	3.3
	Not more than 1 h/day	9	9.8
	2–3 h/day	31	33.7
	More than 3 h/day	49	53.3

### The perspectives of participants about telenursing and their knowledge

Nursing students have not previously had their attitudes and knowledge about telenursing evaluated. The findings of our study persuade us that advancement in education as a whole can increase nursing students’ acceptance of telenursing. The knowledge, opinions, and awareness about future employment that students possess are significantly influenced by their education [4, 9]. Moreover, the majority of participants 76 (82.6%), struggled to accurately define the term "telenursing," 82 (89.1%) of them answered "telemedicine" in the erroneous way (Table 2).

**Table 2:** Participants’ knowledge of telenursing (N = 92).

Variable	Response	Frequency	Percentage
Definition of telemedicine	Right answer	82	89.1
	Wrong answer	10	10.9
Definition of telenursing	Right answer	76	82.6
	Wrong answer	16	17.4

Further, participants were aware that practically using the internet 68 (73.9%), mobile phones 58 (63.0%), audio-video conferencing systems 55 (59.8%), tele-electrocardiograms (ECG) 51 (55.2%), tablets 49 (53.3%), and tele robotics 48 (52.1%) is required for telenursing practice. However, the landline phone 49 (53.3%), and TV 42 (45.6%) was difficult to determine, according to the participants.

According to the participants’ perceptions of telenursing, the majority agreed that telemedicine services should be added to the healthcare system in our country (54.3%), telenursing is an additional patient care option for their future employment (53.3%), and including telenursing in undergraduate curricula would be beneficial for future healthcare professionals (43.5%) (Table 3).

**Table 3:** Perceptions of participants toward telenursing (N = 92).

Variable	Strongly agree/agree		Difficult to say		Strongly disagree/disagree	
	Frequency	Percentage	Frequency	Percentage	Frequency	Percentage
Do you think that telenursing in undergraduate studies would be useful for future healthcare workers?	40	43.5	20	21.7	32	34.8
Would you like to use telenursing as an additional form of patient care in your future work?	49	53.3	16	17.4	27	29.3
How do you assess the need to introduce telemedicine services in the healthcare of your country?	50	54.3	26	28.3	16	17.4

### Applications of telenursing in different nursing specializations and predictors

Most participants (60.8%) agreed that telenursing can be most widely used in diabetes nursing, followed by cardiac nursing (57.6%), pediatric nursing (56.5%), long-term nursing (55.4%), pulmonary nursing (53.3%), and environmental health nursing (52.2%). Pearson correlation coefficient was used to examine the association between students’ perceptions of using telenursing in patient care and their age, gender, access to computers and the internet, frequency of using the internet, and telenursing knowledge. The correlation between a student’s age (r = 0.35), frequency of using internet (r = 0.41), and knowledge of telenursing (r = 0.34) was shown to be moderately significant and positive.

When it comes to how students perceive telenursing, there is a slight significant positive association (r = 0.26, P **<** 0.05) between their gender. After further study using independent t-tests to compare gender differences, it was found that there were no significant differences between the genders (P = 0.41).

There is a weak positive but statistically significant association between students’ perceptions of telenursing and their gender. This can be explained by the fact that telenursing education and training were frequently unavailable to nursing students. This broad unsatisfactory experience and information could result in the same perceptions regardless of gender [22]. In contrast to gender, individual characteristics, experiences, or personal views may have a greater impact on how telenursing is perceived. Other elements may have a greater influence on views than gender alone, such as earlier technological experiences, individual attitudes regarding remote healthcare delivery, or familiarity with technology [20, 21]. Additionally, the particular sample of nursing students used in the study may have an impact on the results that were found. There may be a weaker correlation between gender and perceptions of telenursing if the sample is varied and contains people with different backgrounds, experiences, or attitudes [29]. As a result, it’s possible that the assessment instruments or scales used to evaluate telenursing perceptions aren’t sensitive enough to detect minute gender variations. A weak observed difference may result if the measurement tool is unable to accurately capture the subtleties of gender-related perceptions [17].

To evaluate the student determinants of perception of using telenursing in patient care, stepwise linear regression analysis was used. Age, internet usage patterns, and telenursing knowledge were taken into consideration. A 32% of the variance was explained by the model (Adj. R^2^ = 0.32, F = 15.331, P < 0.05) (Table 4).

**Table 4:** Summary table of multiple regression analysis of telenursing perception predictors (N = 92).

Predictors	B	β	t	F	R ^2^	Adjusted R ^2^
Age	3.543	0.283	3.976			
Internet usage patterns	0.944	0.394	2.817			
Telenursing knowledge	0.853	0.232	1.431	15.331*	0.332	0.320

* p < 0.05

Age, internet use frequency, and knowledge of telenursing were the main factors that substantially predicted telenursing perception, with internet use frequency accounting for 12.9% of the variance. Age and telenursing knowledge were each accounted for in turn (10% and 9.1%).

## Discussion

The purpose of the current study was to assess nursing students ’perception, and knowledge about telenursing. The findings revealed a lack of awareness among nursing students on the definition of telenursing and the prerequisites for practicing it, as well as positive attitudes toward the practice.. The majority of them thought it was necessary to include telenursing concepts in the undergraduate curriculum. Three factors, including age, frequency of internet use, and knowledge with telenursing, were found to have an impact on how telenursing is seen to be used in patient care.

The findings of this study are in line with several investigations into nursing students’ perceptions of the use of telenursing in patient care [8, 20, 21, 30]. Contrary to past research, this research reveals that participants’ perceptions regarding using telenursing to deliver patient care were significantly influenced by factors like age, frequency of internet use, and knowledge of the practice [9, 15]. The most likely explanation is that the majority of students had smartphones, and their age range ranged from 20 to 30. Additionally, a majority of nursing students (97.8%) had access to the internet and used it for more than three hours each day (53.3%).

Moreover, the findings of the study demonstrated that nursing students have insufficient knowledge about the telenursing. The majority of the students were unable to accurately identify the definition of telemedicine as opposed to telenursing, our results were consistent with those of the past studies [9, 29]. These results differed from those of an earlier study [15, 31]. These results could be explained by the possibility that neither teachers nor students received even a basic presentation on the expanded roles of the nursing profession. Accordingly, these findings emphasize the value of teaching nursing students about telenursing in particular.

Further, the results also show that nursing students have positive attitudes toward telenursing despite the lack of awareness. This is good since a foundation of positive attitudes can support additional learning and growth in this area. These students might improve their telenursing skills and aid in its widespread acceptance in healthcare settings with the right instruction and training [32]. Also, the nursing students’ enthusiasm for the use of telenursing for remote monitoring, accessing health information, and follow-up in different specialties of chronic diseases for adults and children in acute, critical, community settings can be explained by the benefits of telenursing use on practice and quality by fostering nurse-patient communication and encouraging positive education outcomes, seamless nursing care, and positive experiences; and [33] a key component of modern nursing education and practice is telenursing.

Numerous studies that have been published have stressed the value of telenursing integration into nursing curricula and the introduction of telenursing services into the healthcare system [17, 19, 22, 32–34]. Similar to this, a sizable majority of nursing students (43.5%) in this study reported that telenursing would be beneficial for future practice and that telenursing services should be implemented in the healthcare system (54.3%) and this consistent with [15, 24]. The positive perspectives of the nursing students highlight the necessity to incorporate telenursing concepts into undergraduate courses to fully equip them to use technology to provide healthcare services to Jordan’s unreached population in response to any impending epidemic emergency situation.

Further, in this survey, the majority of the participants reported that telenursing made it easier for nurses to interact with patients, increased nurses’ productivity (54.4%), and indirectly decreased patient care costs (53.3%). Similar results were seen in earlier research involving undergraduate nursing students [15, 29]. Along with, the nursing students also disagreed that telenursing can result in technological problems (54.4%) and lose direct contact with the medical staff and the patient (45.7%). These results differed from earlier studies in which the majority of nursing students highlighted that telenursing could result in technical problems, lose direct contact between the nurses and the patient, increase the likelihood of mistakes made by nurses, and be inconvenient for patients who are being cared for directly and are unfamiliar with modern technologies [9, 29]. So far, the widely held of nursing students believed that telenursing can be used in all health specialties, which is consistent with past studies [9, 29, 35].

Furthermore, an international survey found that 89% of nurses thought that basic nursing education should include telenursing, and the authors came to the conclusion that telehealth education should also involve clinical experiences [1]. Additionally, published data [15, 36, 37] also point to a favorable shift in nursing students’ opinions following the implementation of telenursing instruction sessions. Nursing programs might think about introducing telenursing concepts into their curricula to alleviate the lack of awareness. This can involve educating people about telenursing theoretically, debating its advantages and disadvantages, and providing opportunity for practical experience in simulated or real-world settings. This will enable nursing students to embrace this new area of nursing practice by giving them a greater understanding of telenursing and its requirements. These results intensely support the necessity for telenursing principles to be included in undergraduate nursing courses. However, further study is required to fully comprehend the most effective telenursing teaching techniques.

## Conclusion

The findings of this study demonstrated that nursing students are enthusiastic about the application of telenursing in practice. However, the majority of them were unfamiliar with the definition of telenursing and telemedicine. The majority of students reported that telenursing was future-focused and that nursing curricula should include telenursing concepts in order to equip future nursing professionals to deliver safe and competent care in a highly technical and digital world.

### Recommendation

To address the core gap surrounding concerns connected to telenursing we recommend nursing students take telenursing-related online courses or modules. Various facets of telenursing, such as definitions, applications, technologies, legal and ethical considerations, and best practices, are covered in these courses, which can offer structured learning [10]. Attend webinars, conferences, or workshops that focus exclusively on telenursing. These gatherings frequently include knowledgeable presenters who may offer in-depth explanations of the definition, application, and most recent developments in telenursing [13]. Additionally, it offers chances to engage in dialogue and networking with industry experts, and get in touch with a mentor or coworker who has telenursing expertise. Also, to increase nursing students’ understanding and clear up any misunderstandings, have discussions with them, ask for their advice, and be explicit in your queries [15]. Moreover, gaining knowledge of telenursing-related issues needs lifelong learning and staying updated with the latest research and practices in the field.

Hence, the study’s findings have implications for several different areas. The study gave new knowledge on the factors influencing telenursing. Informatics education curricula should be assessed for their content and efficacy. With a greater focus on the telenursing principles, it is crucial in nursing education to create educational and training programs that fit each student’s unique needs and clinical practice requirements. For the dynamic growth of patient care via telenursing in Jordan, the notion of telenursing must also be introduced into the undergraduate curriculum. The study highlights the areas that nursing faculty should pay close attention to. Future research using a random sample is required, as well as an interventional study to assess the effectiveness of the offered courses using an experimental approach.

### Limitations

The study’s limitations include the convenience sample’s restriction to two nongovernmental colleges, each of which has different resources and capabilities, which may limit the study’s generalizability. The survey respondents’ understanding may not exactly mirror that of the non-respondents. Also, the use of a self-report questionnaire could introduce bias since participants might not always give accurate descriptions of their perceptions of telenursing. Additionally, only nursing students in their third and fourth years were included in the sample. As well, we were unable to monitor the attitude changes among nursing students from various academic years and governmental universities that offer nursing programs. Consequently, the findings of this study have a limited degree of external validity.

## Supporting information

S1 Data (XLSX)
